# Signaling in Legume–Rhizobia Symbiosis

**DOI:** 10.3390/ijms242417397

**Published:** 2023-12-12

**Authors:** Julia Shumilina, Alena Soboleva, Evgeny Abakumov, Oksana Y. Shtark, Vladimir A. Zhukov, Andrej Frolov

**Affiliations:** 1 Laboratory of Analytical Biochemistry and Biotechnology, Timiryazev Institute of Plant Physiology, Russian Academy of Sciences, 127276 Moscow, Russia; schumilina.u@yandex.ru (J.S.); soboleva@ifr.moscow (A.S.); 2 Biological Faculty, Saint Petersburg State University, 199034 St. Petersburg, Russia; e_abakumov@mail.ru; 3 Laboratory of Genetics of Plant-Microbe Interactions, All-Russia Research Institute for Agricultural Microbiology, 196608 St. Petersburg, Russia; oshtark@arriam.ru (O.Y.S.); vzhukov@arriam.ru (V.A.Z.)

**Keywords:** legume–rhizobia symbiosis, nitrogen fixation, infection, regulation, signaling, determinate and indeterminate nodules, nodule senescence

## Abstract

Legumes represent an important source of food protein for human nutrition and animal feed. Therefore, sustainable production of legume crops is an issue of global importance. It is well-known that legume-rhizobia symbiosis allows an increase in the productivity and resilience of legume crops. The efficiency of this mutualistic association strongly depends on precise regulation of the complex interactions between plant and rhizobia. Their molecular dialogue represents a complex multi-staged process, each step of which is critically important for the overall success of the symbiosis. In particular, understanding the details of the molecular mechanisms behind the nodule formation and functioning might give access to new legume cultivars with improved crop productivity. Therefore, here we provide a comprehensive literature overview on the dynamics of the signaling network underlying the development of the legume-rhizobia symbiosis. Thereby, we pay special attention to the new findings in the field, as well as the principal directions of the current and prospective research. For this, here we comprehensively address the principal signaling events involved in the nodule inception, development, functioning, and senescence.

## 1. Introduction

Legumes represent one of the most accessible sources of food protein and, therefore, essentially impact on the human diet. Importantly, the productivity of legume crops strongly depends on the availability of macronutrients. Among them, nitrogen represents one of the most naturally abundant elements, which is vitally essential for plants [1]. However, only a minor part of the overall nitrogen pool can be found as nitrates and ammonium nitrogen, which have high solubility in water and can be readily absorbed by roots. Thus, the deficiency of metabolically available nitrogen in soils is one of the principal factors limiting their productivity [2]. On the other hand, the largest part of the global nitrogen pool is represented by its highly stable and metabolically inactive atmospheric molecular form, which is unavailable for plants.

Fortunately, due to their ability to fix atmospheric nitrogen, rhizobia (a group of soil Gram-negative bacteria from the phylum *Proteobacteria*) provide an additional source of metabolically available nitrogen and essentially facilitate uptake of this macronutrient by plants. Free-living rhizobia are able to infect plant roots and form characteristic morphological structures known as root nodules. The resulting legume–rhizobia symbiosis is usually referred to as a plant–microbial interaction between the roots of legume plants and rhizobia [3]. Therefore, growth, stress tolerance, and field yields of legume crops directly depend on the success of this symbiosis [4]. In this context, deep and comprehensive understanding of the mechanisms behind the onset and development of legume–rhizobia symbiosis is absolutely mandatory to increase productivity of the legume crops.

**Figure 1:**
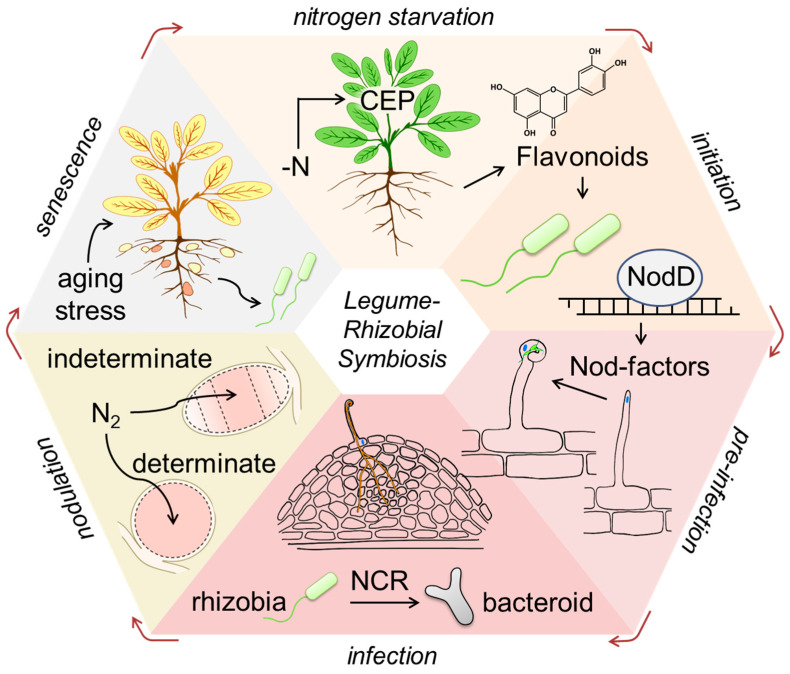
The stages of the legume–rhizobia symbiosis. CEP—C-terminally encoded peptide; NodD—nodulation protein D; NCR—nodule-specific cysteine-rich peptides; N or N_2_—nitrogen.

To date, the legume–rhizobia symbiosis is well-characterized, and the underlying mechanisms of genome regulation represent the most comprehensively elaborated aspect of this phenomenon [5,6,7,8,9,10]. Therefore, the individual steps of the corresponding regulatory pathways were addressed in much detail [11,12,13,14]. The principal signaling events coordinating the development of the legume–rhizobia symbiosis come into play sequentially. Nodulation assumes a coordinated progress of bacterial infection and nodule organogenesis, i.e., two processes that are common for a broad range of legume species [15]. At the initial step, the plant attracts rhizobia by enhanced root excretion of flavonoids and isoflavonoids [3]. In turn, after perception of the flavonoid signal, rhizobia synthesize and secrete so-called nodulation factors (Nod factors), which penetrate the epidermis of the plant’s roots and thereby trigger the infection process [16,17,18]. This step is manifested by the formation of the infection thread, which grows through the root cortex to reach the *de novo* developed organ—the nodule primordium—formed as a result of the division of the newly emerged meristematic cells [19]. At the next step, the rhizobia penetrate the cells of the nodule primordium. From this time point, the primordium can be considered as a nodule, whereas rhizobia differentiate into bacteroids [20]. Depending on the ontogenetic dynamics of their meristematic activity, nodules can be classified as determinate or indeterminate [20]. Independently from their type, the further development and metabolism of the nodule are primarily controlled by the host plant. Therefore, the onset and the progress of nodule senescence are also coordinated by the legume plant (Figure 1). Obviously, the overall success of the symbiosis and its long-term efficiency strongly depend on multiple regulatory aspects at each step.

However, although legume–rhizobia symbiosis has been studied for several hundred years [21], and an impressive body of information has been successfully collected so far, the knowledge about the fine mechanisms underlying the molecular dialogue between the partners and related signaling events is still incomplete and is being continuously extended. Thus, several critically important signaling aspects of the plant–rhizobia interaction, like the possible role of CEPs (C-terminally encoded peptides) in the induction of flavonoid biosynthesis, flavonoid-mediated NodD activation, perception of Nod factors by plants, regulation of the infection thread growth, rhizobium differentiation, regulation and coordination of nodule senescence, still remain insufficiently addressed in literature [22,23,24] and need to be studied in more detail to gain understanding of the fine mechanisms underneath. Understanding the details of the molecular mechanisms behind nodule formation and function might give access to new legume cultivars with improved crop productivity. In particular, obtaining mutants with more efficient legume–rhizobia symbiosis might result in a pronounced increase in plant stress tolerance and productivity.

Therefore, in this review, we comprehensively address the signaling aspects of all critical steps of the nodule development: mutual recognition, flavonoids as attractants for rhizobia, nodulation protein D (NodD) and its activation, rhizobial Nod factors (NFs) and their perception by plants, NF-independent initiation of symbiosis, the symbiosis signaling pathway (that is also shared with another endosymbiosis, arbuscular mycorrhiza), terminal bacteroid differentiation governed by nodule-specific cysteine-rich peptides (NCR), autoregulation of nodulation, nodule senescence, and the effect of the environmental stress on this process (Figure 1). Thereby, a special emphasis is given to the aspects most intensively addressed during the last decade, i.e., which can be judged as the boundaries of the research in the field of plant–rhizobia symbiosis.

## 2. Mutual Recognition: Flavonoids as the Attractants for Rhizobia

**Figure 2:**
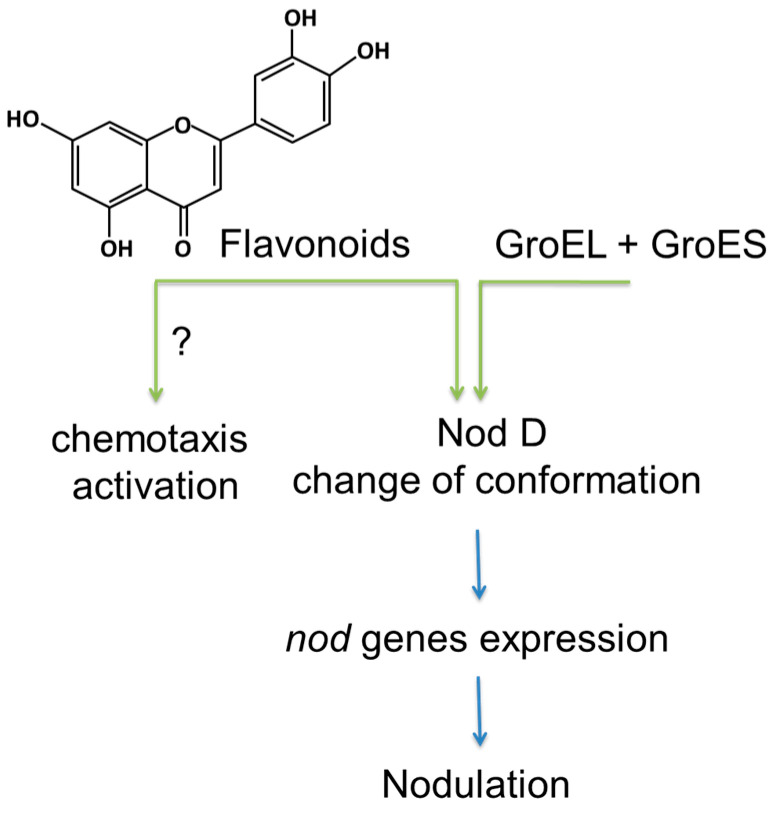
The role of flavonoids in nodulation and establishment of legume–rhizobia symbiosis. NodD—nodulation protein D; GroEL and GroES—chaperonins, which are the homologs of the eukaryotic heat shock proteins 60 and 10 kDa (HSP60 and HSP10), respectively.

Perception of nitrogen deficiency and generation of the nitrogen starvation signal are accompanied by attraction of rhizobia to the plant root. This step, in turn, relies on the synthesis and excretion of flavonoids, which act as the signaling molecules secreted by root cells [25,26] (Figure 2). These polyphenols probably belong to the group of chemoattractants for rhizobia, which also include amino acids—e.g., glutamate, proline, arginine, phenylalanine, tryptophan [27,28], and carboxylates, like citrate, malate, and succinate [29,30]. Importantly, flavonoids activate an array of genes in rhizobial cells, which encode enzymes involved in the synthesis of rhizobial Nod factors [31,32].

Flavonoids represent a diverse structural class of polyphenolic compounds universally spread in all organs and tissues of plants [33]. Due to their pronounced antioxidant, anti-inflammatory, and anticarcinogenic activities in humans, these polyphenols attract special attention [34]. Flavonoids constitute several structural sub-classes—chalcones, flavones, isoflavones, flavonols, and anthocyanins [34]. In plants, flavonoids are involved in protection against abiotic and biotic stress (flavones, flavonols), reproduction (isoflavones), and tissue pigmentation (flavonols, anthocyanins) [35,36]. 

Occurrence of flavonoids in the rhizosphere can be explained by two principal mechanisms. On one hand, these polyphenols can be released from decomposing root sheath and border cells. On the other hand, their excretion in the environment can be triggered by nitrogen starvation and might be released by transporters [37,38]. For example, Sugiyama and co-workers showed that the release of genistein relied on the activity of ATP-dependent ABC transporters [39]. It is known that ABC transporters demonstrate higher affinity to glycosides in comparison to aglycons [35]. Therefore, secretion of glycosides is more efficient in comparison to the corresponding aglycons [40] and is, hence, more likely. This observation is in line with chemical properties of flavonoid glycosides, which, due to their higher (in comparison to corresponding aglycons) solubility, are more efficiently exported to the environment in comparison to their non-glycosylated forms [41]. On the other hand, the glycosides might be readily deglycosylated by glycosidases, which are constitutively expressed by soil microorganisms [42]. This is consistent with the fact that the amounts of aglycons are higher in root exudates in comparison to glycosides [42]. 

On the other hand, Biała-Leonhard and co-workers showed an important role of the plasma membrane-localized MATE-type transporter in the release of isoflavonoid genistein from white lupin roots [38]. They reported, that the LaMATE2 expression was up-regulated in the root under the conditions of nitrogen and phosphate deficiency, and LaMATE2 silencing reduced genistein efflux and even more the formation of symbiotic nodules. This provide a possible role of LaMATE2 in isoflavonoid release and nodulation. Thus, a drop in amounts of the metabolically available soil nitrogen might result in an increased level of flavonoid biosynthesis, and the expression of the genes encoding a flavonoid transporters [38,43,44]. It highlights nitrogen starvation as the factor promoting flavonoid synthesis in roots of legumes (Figure 2).

Despite the fact that plants constitutively synthesize a broad selection of flavonoids and their derivatives, relatively few aglycons could be identified in root exudates (and can be considered, therefore, as candidates for signaling players). Thus, to date, this list is mostly restricted to aglycons of luteolin, hesperetin, genistein, naringenin, genistein, and daidzein with only minor contribution of other substances [45]. It needs to be taken into account that increase of the flavonoid contents in root exudates typically accompanies stress-induced metabolic adjustment [46]. Thus, attraction of rhizobial symbionts might be a physiological adaptation behind the stress tolerance of legume plants [47].

Flavonoids are differentially distributed in root tissues, accumulating predominantly in proliferating cells, i.e., in actively growing root parts [48]. In agreement with this, these polyphenols were shown to be abundant in lateral roots and nodule primordia of subterranean clover (*Trifolium subterraneum* L.) and in the root tips and lateral root primordia of Arabidopsis (*Arabidopsis thaliana* (L.) Heynh.) [49,50]. On the other hand, Nouwen et al. showed that supplementation of legume root exudates (from *Aeschynomene afraspera* J. Léonard) and pure naringenin in the growth medium led to a significant increase in the density of the rhizobial culture (*Bradyrhizobium* sp.) [41]. Later on, the same group demonstrated up to four-fold dose-dependent changes in the density of the bacterial culture induced by supplementation of naringenin to the medium [51]. Importantly, the authors found that the growth-promoting effect was independent from the regulators of the *nod* genes encoding the enzymes involved in the biosynthesis of Nod factors [51]. Thus, these works demonstrated involvement of flavonoids in the regulation of growth dynamics of the rhizobial culture. This assumes the existence of a distinct molecular mechanism affecting rhizobial populations *in vivo*, although its nature remains unknown (Figure 2). The specificity of flavonoids as attractants for specific species and/or strains of rhizobia was also highlighted, or acting as a phytoalexin and eliciting antimicrobial activity [52]. Nevertheless Compton et al. propose that flavonoids play a significant role in *S. meliloti* chemotaxis is insignificant relative to other components released by alfalfa seeds [53]. Taken together, the evidence for flavonoid chemotaxis as a general phenomenon in rhizobia is debatable [3]. 

Remarkably, multiple flavonoids can play both stimulating and inhibitory roles in the development of symbiosis with arbuscular mycorrhizal fungi. In particular, low concentrations (a few µmol/L) of flavonoids appear to be able to stimulate germination of fungal spores, as well as enhanced elongation of their hyphae and prolonged asymbiotic growth, thereby increasing the physical distance and time period for possible contact with the host. Thus, flavonoids impact on the establishment of these two agriculturally important root symbioses—nodulation and mycorrhizal interactions [54,55].

## 3. Mutual Recognition: Nodulation Protein D (NodD) and Its Activation

The mechanistic aspects behind the activation of bacterial *nod* genes by exogenic flavonoids are not yet fully understood. In contrast, it is well known that expression of the *nod* genes is controlled by the specific transcription regulator known as NodD (nodulation protein D) [56]. NodD belongs to the LysR family of transcription activators, which are the proteins of approximately 35 kDa with a characteristic N-terminal helix-turn-helix DNA-binding domain [57]. These activators are often repressed and usually require inducers for activation. Some rhizobial species express SyrM protein, which is a homolog of NodD. In the rhizobial cell, NodD is represented by three isoforms—NodD1, NodD2, and NodD3—which are highly conserved and characterized by a high degree of homology to each other [52]. Moreover, multiple species have several copies for each of the *nod* genes [52]. For example, the genome of *Sinorhizobium meliloti* contains all three types of NodD isoforms, which are more than 77% identical in the amino acid sequence [58]. Interestingly, despite this sequence similarity, these three NodD isoforms are physiologically not equivalent. Thus, activation of NodD1 and NodD2 requires plant-derived polyphenolic inducers, whereas NodD3 can be expressed even in the absence of flavonoids [57]. Most likely, upon binding to the inducer, the NodD protein acquires affinity to a highly conserved 55 nucleotide pair sequence in the promoters of the *nod* genes, usually referred to as the *nod* box. This interaction triggers expression of *nod* genes [57].

Unfortunately, there is no unambiguous evidence for direct binding of flavonoids to the NodD regulator, although multiple attempts to identify such an interaction have been made during recent decades. Thus, Peck and co-workers addressed the role of flavonoids as direct regulators of NodD activation in *Sinorhizobium meliloti* culture *in vitro*. The authors showed that the affinity of NodD1 to the *nod* gene region increased three- to four-fold in comparison to the control in the presence of GroEL protein and luteolin [57]. The authors hypothesized that binding of NodD1 protein to flavonoids (such as luteolin, eriodictyol, daidzein, 7-hydroxyflavone, and naringenin) might be triggered by the changes in its conformation. Most likely, this binding requires interaction with GroEL and might directly contribute to the affinity of the NodD1 protein to the promoter region of *nod* genes (Figure 2). Competitive binding analysis, accomplished with a broad selection of flavonoids (naringenin, eriodictyol, luteolin, and daidzein), unambiguously demonstrated that non-inducing flavonoids (i.e., those not affecting expression of the *nod* genes) act as competitive inhibitors of luteolin, whereas only luteolin is able to trigger NodD activation, necessary for the induction of the *nod* genes of *Sinorhizobium meliloti* [57].

One needs to take into account, however, that different flavonoid ligands might affect conformation of the NodD protein in different ways [59]. In this context, discovery of the structure-activity relationships (SAR), i.e., functional relations between the structures of the signaling polyphenols and their regulatory effects, appears to be an important step forward. Thus, after comprehensive functional analysis accomplished with a broad range of flavonoids, Cooper reviewed a clear correlation between the structures of the polyphenols and their potential for induction or suppression of the nodulation genes [26]. According to his study, independently from the other ring substitutions, the hydroxyl groups at the positions 7- and/or 4′ appeared to be critically important for initiation of the nodules. For example, expression of the *nod* genes in *Rhizobium leguminosarum* bv. *viciae* was shown to be enhanced by flavonoids having hydroxyl groups at the 4′-, 5-, and 7-positions [26].

Unfortunately, the fine mechanistic aspects of NodD activation addressed for a few rhizobial species so far. For example, involvement of GroEL in the induction of *nod* genes was demonstrated solely for *Sinorhizobium meliloti* [60]. It also needs to be mentioned that the transport of flavonoids into the rhizobial cell and the mechanisms controlling their further interaction with NodDs are just minimally addressed so far in other rhizobial species besides *S. meliloti*. Thus, extending the mechanistic molecular studies to other species and understanding the general principles of NodD functioning might be the main road of further studies in this direction.

## 4. Initiation of Legume–Rhizobia Symbiosis

Nod factors (NFs) represent the class of signaling molecules containing *N*-acetylglucosamine-rich oligosaccharides as the main core with different side-chain modification groups—predominantly alkyl, acetyl, arabinosyl, fucosyl, glycerol, and sulphate substitutions [61,62,63]. The functional effects of NFs are manifested as induction of root hair deformation, proliferation of root cortical cells, and formation of infection threads [15]. Different nodulation efficiency of two strains within the same species might be underlied by the structures and relative abundances of individual NFs synthesized by each of them [64,65]. This fact might explain why some strains are more successful in forming symbiotic relationships than others.

The biosynthesis of this generalized NF core relies on glucosamine synthase (NodM), *N*-acetyl glucosamine transferase (NodC), deacetylase (NodB), and acyl transferase (NodA) [66]. At the first step, glucosamine-6-phosphate is synthesized from fructose-6-phosphate in the reaction catalyzed by glucosamine synthase with formation of *N*-acetylglycosamine. Then, the resulting *N*-acetyl-glucosamine undergoes oligomerization yielding molecules containing three to five *N*-acetyl-glucosamine monomers. At the next step, the fatty acid (FA) residue is attached to the oligo-*N*-acetylglucosamine chain by an appropriate acyltransferase [66]. Typically, the FA component of NFs is represented by mono- or polyunsaturated C16 or longer chains [67]. For example, the NFs of *R. meliloti* contain C16:2 and C16:3 FA moieties, while the NFs isolated from *R. leguminosarum* bv. *viciae* have various C16, C18:2, C18:3, and C18:4 residues [68,69]. This might indicate that the species-specific FAs are employed in the NF synthesis. 

After completion of their biosynthesis, NFs are released from the rhizobial cell via membrane transporters, which are also encoded by *nod* genes [26]. The secreted NFs bind to their receptors on the surface of plant epidermal cells. The activation of the receptors triggers a plant signaling cascade containing SYMRK, CCaMK, CYCLOPS, NIN, and other proteins. As many these proteins are also involved in the so-called common symbiotic signaling pathway, some of them belong to the arbuscular mycorrhizal signaling. This suggests that the signaling pathway initially functioned in mycorrhizal signaling and was later recruited in an ancestor of the legumes for recognition of rhizobia [15,70] The mechanisms that drove this evolutionary step have become clearer with the discovery that arbuscular mycorrhizal fungi produce NF-like molecules [71].

**Figure 3:**
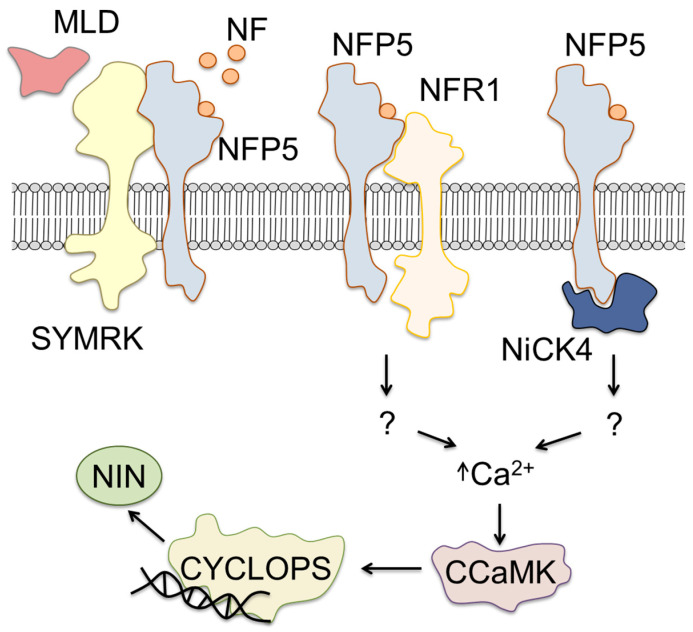
Signaling pathways in the root cells activated by Nod factors. MLD—malectin-like domain; SYMRK—LRR-containing receptor kinase; NF—Nod factors; NFR1 and NFR5—Nod factor receptors 1 and 5; NiCK4—NFR5-interacting cytoplasmic kinase 4; CCaMK—calcium/calmodulin-dependent serine/threonine-protein kinase; NIN—nodulation inception transcription factor.

Activation of the symbiotic signaling cascade enhances the growth of root tissues and facilitates penetration of rhizobia in the root cells (Figure 3) [72]. Thereby, it is important to note that initiation of the root nodules does not occur in random locations on the root surface but shows clearly characteristic distribution. Specifically, Bhuvaneswari and co-workers identified the site of possible rhizobial infection and nodulation as the region of the root, which corresponded to the extension and differentiation zone [73].

Most likely, due to increased permeability of cell walls in the zone of the extension growth, Nod factors successfully interact with their receptors, which are represented by serine/threonine kinases of the LysM type [70]. For the first time, the receptors for Nod factors were identified in *Lotus japonicus* (Regel.) K. Larsen (NFR1, NFR5) [74] and *Medicago truncatula* Gaertn. (LYK3, NFP) [75]. After that, the homologs of the *NFR1*/*LYK3* and *NFR5/NFP* genes were found in other legumes such as *Pisum sativum* L. (K1, SYM10, SYM37, LykX) [76,77,78,79].

Currently, the most well-characterized receptors for Nod factors are NFR1 and NFR5, identified in *L. japonicus*. These molecules are the transmembrane integral proteins localized in the plasma membrane of root epithelial cells [80]. The characteristic NF-binding sites are localized in their extracellular domains and typically demonstrate broad specificity for rhizobial Nod factors [80,81]. To date, the signaling pathways triggered by activation of these receptors are, at least partly, deciphered. Thus, in the absence of symbiosis, the malectin-like domain (MLD) dissociates from the LRR-containing receptor kinase (SYMRK) [72] (Figure 3). In this dissociated state, SYMRK is able to interact with NFR5 and changes, thereby, the conformation of this receptor [72]. This event affects the conformation of the receptor in the way facilitating attachment of a Nod factor. After the complex of the Nod factor with NFR5 is formed, the kinase most likely triggers the reversal conformation change.

It is important to note that, in contrast to NFR1, NFR5 is a pseudokinase, i.e., it does not have an intracellular kinase domain for the signal transduction [72]. Therefore, depending on the availability of NFR1 for interaction with the activation partner, NFR5 might result in at least two possible ways (Figure 3). According to the first way, NFR1 (which has kinase activity) forms a heterodimer with NFR5, facilitating further signal transduction [82].

Alternatively, in the absence of the NFR1 partner, NFR5 might bind to NFR5-interacting cytoplasmic kinase 4 (NiCK 4) [83]. Interestingly, NiCK4 was found to catalyze phosphorylation of the intracellular domain of NFR5. Wong et al. proposed that phosphorylation of NFR5 makes its regulatory sites available for interaction with further messengers and triggers, thereby, signal transduction [83]. It cannot be excluded that NFR1 is also able to phosphorylate NFR5 in the same way. The activation of this pathway triggers release of calcium ions from the intracellular depot. This Ca release activates calcium/calmodulin-dependent serine/threonine-protein kinase (CCaMK) (knows as DMI3 in *M. truncatula*, for Does not Make Infections 3), which phosphorylates the transcription factor CYCLOPS (IPD3, for Interacting Protein of DMI3 in *M. truncatula*) [84]. At the next step, CYCLOPS induces expression of the nodulation inception transcription factor (NIN), that activates symbiotic genes [82,84] (Figure 3). In *Medicago truncatula*, DELLA proteins promote phosphorylation of IPD3 (protein CYCLOPS of *M. truncatula*) and interactions between IPD3 and NSP2/NSP1 (nodulation signaling pathway protein 1 and 2), which bind to the promoter sequence of NIN and thereby activate its expression [85,86,87].

Remarkably, pea (*Pisum sativum* L.) represents a unique example of an extremely high specificity of the interaction between the rhizobial and legume partners during formation of the nodule symbiosis. For example, the pea cultivars originated from Afghanistan and Iran appeared to be unable to form nodules with the majority of the natural *Rhizobium leguminosarum* bv. *viciae* strains isolated from European soils. On the other hand, these pea cultivars readily and efficiently interact with the strains from the Middle East. Among the latter, *R. leguminosarum* strain TOM, which has a *nodX* gene, encoding a specific O-acetyltransferase modifying the reducing end of the sugar backbone in NFs, can be mentioned. This feature of the pea plant is controlled by a specific recessive allele of *Sym2* gene usually referred to as the “Afghan allele” (*Sym2^A^*) [10]. Although this was the first pea symbiotic gene to be discovered, its molecular function is still elusive. The recently discovered pea gene *LykX* (LysM kinase exclusive) is a promising candidate for *Sym2* [79]. According to the current state of the knowledge, the high specificity of the inter-partner interaction in the legume–rhizobia symbiosis emerged independently at least twice during the evolution of pea. This fact might highlight the importance of this phenomenon for the pea [88]. 

Although it is generally believed that the Nod factors are the critical players in the curling of the root hair tips around the rhizobia with the formation of an infection chamber [89,90], their presence is not sufficient for initiation of the infection. Indeed, bacterial exopolysaccharides (i.e., the polymers constituting the cell walls of the microorganisms) need be involved as well [91,92]. This fact assumes the necessity of the direct contact between the bacterial cell and its plant counterpart for successful infection. This assumption was experimentally confirmed by Cheng and Walker, who demonstrated the failure in the infection by a *Rhizobium meliloti* mutant defect by the gene *exoY*, which is responsible for the synthesis of succinoglycan—a mechanical exopolysaccharide of the rhizobial cell [93]. Thus, it can be concluded that, despite the critical role of the plant in the regulation of the rhizobial infection (i.e., penetration of the root epidermis cell by the microorganism), the feedback of the bacterial partner at this step is important for successful onset of the symbiosis.

The perception of rhizobial exopolysaccharides has been studied in the most detail using the example of *L. japonicus.* It was found that infection thread growth and cell infection in this plant are provided by recognition of compatible rhizobia surface exopolysaccharides by the host’s trans-membrane lysin motif (LysM) receptor kinase EXOPOLYSACHARIDE RECEPTOR 3 (EPR3) [94,95]. This protein has a special configuration of three LysM domains (LysM1-LysM2-LysM3) due to the atypical topology of LysM1 [94,96]. Thereby, the extracellular domain of EPR3 is specific to the exopolysaccharide structure and does not bind to fungal and rhizobia chitooligosaccharide and lipo-chitooligosaccharide signal molecules [96]. Thus, EPR3 works as a secondary identity-based mechanism in the establishment of nitrogen-fixing nodule symbiosis between *L. japonicus* and its microsymbiont *Mesorhizobium loti.* Studies on EPR3-type LysM receptors in species other than *L. japonicus* are limited. In *Medicago truncatula*, the *EPR3* ortholog *MtLYK10* is crucial for the infection thread progression to the nodule primordia. However, a protein responsible for recognition of succinoglycan (the surface exopolysaccharide of the *M. truncatula*-compatible microsymbiont *Sinorhizobium meliloti*) was not found [97].

Other auxiliary mechanisms contributing to the infection are NF-independent initiation of the legume–rhizobia symbiosis. Recently, Okazaki et al. confirmed that *nod* genes knockout mutants are also able to induce nodulation [98]. The type III and IV secretory systems (T3SS, T4SS) are responsible for the formation of the nodule symbiosis [99,100]. To date, the role of T3SS in the formation of nodule symbiosis is understood in much detail. Earlier, this system was characterized as one of the main players in the infection of plant roots with pathogenic bacteria, and only recently its role in the symbiosis initiation was described [100,101]. Importantly, two additional factors are necessary for the initiation of symbiosis via T3SS: (*i*) triggering of root rhizobial infection by plant-derived flavonoids and (*ii*) the presence of the NodD protein (which also initiates the transcription of the *nopL* gene involved in the biosynthesis of the T3SS) [102]. This might indicate that flavonoids activate the NodD protein, which, in turn, triggers expression of the *nopL* gene [102,103]. Its product NopL initiates expression of the type III secretory system genes. As this system assumes the direct contact of the signal transducing molecules for infection, the corresponding effectors are delivered directly to the surface of the root cells. These effectors are known as nodulation outer proteins (Nop). Nop represent the rhizobial instrument to affect the host cell metabolism for suppression of plant defense responses and stimulate symbiosis-related processes. Accordingly, the mutant strains deficient in the synthesis of these effectors or defect by the secretion mechanism show a reduced potential to form symbiosis with corresponding legume species [104]. For example, *Sinorhizobium fredii* is known to secrete eight Nop proteins (NopA—D, NopL, NopM, NopP, and NopX), which are the extracellular components of the type III secretory system [105,106,107], via T3SS in response to stimulation with genistein secreted by plant roots [108]. In total, about 30 different Nop proteins were discovered over the last two decades [109]. The most of them were predicted in silico based on the results of transcriptomics analysis, whereas only a few could be characterized experimentally at the level of functional proteins.

Over recent years, more information about the rhizobia, relying on both infection mechanisms, has appeared. Thus, the phenomenon of rhizobial synergy, i.e., improved efficiency of nodule formation as a result of inoculation with several rhizobial strains, was reported [110,111]. 

## 5. Signaling Regulating Rhizobial Infection and Organogenesis

After penetration into the root, one or several bacterial cells get surrounded by the plasma membrane of the host plant cell, forming the infection chamber [112,113]. Depending on the host plant species, one (e.g., *Medicago sativa*) or several (e.g., *Glycine max* (L.) Merr. or *Vigna sinensis* (L.) Savi ex Hausskn.) bacterial cells can be enclosed within each infection chamber [114,115,116,117,118], which is further modified to a so-called infection thread (IT) [119]. IT is usually defined as a tubular structure built by the plasma membrane of the root hair epidermis cells [89]. The enclosed cytoplasm forms the matrix in which the bacteria move during the expansion of the IT [89,113,120]. Importantly, this process is accompanied with the continuous synthesis and exocytosis of the plant plasma membrane and cell wall components [113]. On the other hand, multiple proteins were identified as involved in IT expansion: a coiled-coil protein required for polar growth (RPG), actin rearrangement proteins, cystathionine-β-synthase-like 1 (CBS1), a putative E3 ligase LUMPY INFECTIONS (LIN)/CERBERUS, and VAPYRIN (VPY) [121,122,123,124,125,126].

**Figure 4:**
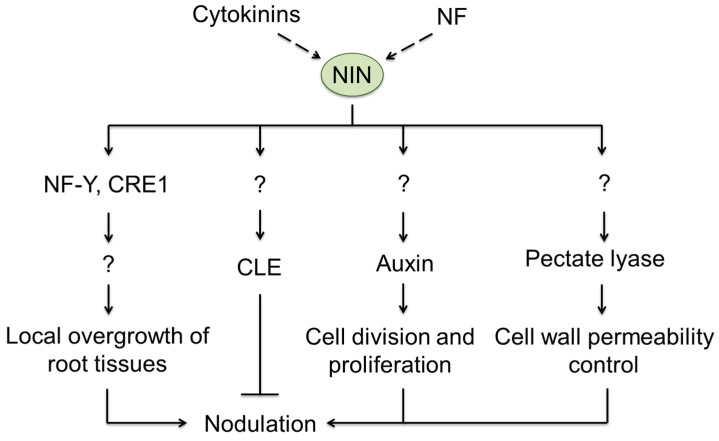
The effects of NIN protein in root cells. NF—Nod factors; NF-Y—nuclear factor-Y; CRE1- cytokinin response kinase 1; CLE—CLAVATA3/Embryo-surrounding region-related peptides.

To date, the molecular mechanisms behind the expansion of IT and release of rhizobia in the cytoplasm of the host cell are, at least partly, identified. The regulatory pathways activated by NIN represent an important part of nodulation signaling. Thus, NIN activates the expression of symbiotic genes, triggering nodulation. Thereby, activation of cell proliferation in response to rhizobial infection is believed to be the primary role of this regulator. Thus, Soyano and co-workers [127] showed that NIN initiates transcription of *NF-YA1* and *NF-YB1* genes in *L. japonicus* roots and synthesis of their products—nuclear factor-Y (NF-Y) family proteins (Figure 4) [127].

The authors also demonstrated that knockout of these genes prevented nodulation, whereas their over-expression correlated with an increase in nodule cell proliferation. This brought the authors to the assumption that these proteins might cause a local explosive proliferative growth of the root tissues, which is required for successful nodule formation [127].

The phytohormones play an important role in the tissue proliferation and nodule formation. In particular, *Medicago truncatula* mutants of the cytokinine receptor gene, *Cytokinine Response 1* (*CRE1*), were featured with increased numbers of lateral roots and a strong decrease in nodulation efficiency [128]. Accordingly, increased levels of cytokinins also enhanced expression of the *NIN* gene. In turn, its product—the NIN protein—was shown to promote a local increase of auxin levels in the root cells, which that also enhanced their proliferation [129] (Figure 4).

Strigolactones are a novel family of phytohormones, which are well-known as positive modulators of arbuscular mycorrhiza [130,131]. Recently, these molecules were also proposed to be involved in nodule formation, although their role in this process is still ambiguous. The genes of strigolactone biosynthesis (*D27*, *CCD7*, and *CCD8*) demonstrated a concerted expression pattern in the nodule primordium, as well as in the meristem of the growing distal part in the *M. truncatula* mature root nodules [132]. It is also known that *P. sativum ccd7* and *ccd8* mutants form reduced numbers of nodules. Moreover, the exogenous treatment of the mutant pea plants with the synthetic strigolactone GR24 was shown to restore the wild type phenotype (i.e., normal nodule numbers) [91,133,134]. However, a recent study accomplished with pea mutants at the genes of strigolactone signaling and biosynthesis (including *CCD7* and *CCD8*) could give some new insights. Thus, the role of GR24 in the delay of the nodule development and senescence was shown. Moreover, these effects are most likely mediated by NIN and available plant sugars [135].

Not less importantly, NIN was reported to enhance the expression of CLAVATA3/Embryo-surrounding region-related peptides (CLE, Figure 4), which are known to be the signals that suppress nodulation [136,137]. Thus, these peptides are involved in regulation of nodule formation and impact on the nodulation efficiency.

In their work with *L. japonicus,* Xie et al. found that NIN protein also impacted the expressional regulation of the gene encoding nodulation pectate lyase (NPL) [138] (Figure 4). Pectate lyase is involved in the control of cell wall permeability and underlies, thereby, the efficiency of rhizobia penetration in the plant root cell [138] (Figure 3 and Figure 4).

Thus, the NIN-controlled signaling pathways affect multiple aspects of the symbiosis: regulation of the nodule numbers, germination and expansion of the infectious thread, penetration of rhizobia in the root cells, and autoregulation of nodulation.

## 6. Terminal Bacteroid Differentiation Governed by NCR Peptides

After penetration into the cytoplasm of root cells, rhizobia undergo transition to bacteroids and formation of symbiosomes [139]. The phenotype of the bacteroids depends on the type of the nodule formed (i.e., determinate or indeterminate) [140,141]. Therefore, this process is controlled by the plant partner [142]. The nodule type is defined at the step of nodule initiation. At the molecular level, definition of the nodule type is underlied by the metabolism of auxins (which are known to enhance proliferation of the root cells) [129]. Accumulation of auxins at the site of rhizobia infection and inhibition of polar auxin transport underlies formation of indeterminate nodules, while formation of determinate nodules is not accompanied with these events [143].

The bacteroids formed in the determinate nodules typically have spherical form, while the bacteroids of the indeterminate nodules show complex branching phenotypes, often referred to as Y-shaped [144]. Despite this morphological difference, both bacteroid types are characterized with a pronounced increase in the area of plasma membrane and cytoplasm volume in comparison to non-differentiated cells [144]. The increase in the contents of bacteroid DNA is associated with the replication of DNA without the subsequent cytokinesis during the maturation of bacteroids [142]. Moreover, the bacteroids of the determinate nodules retain their proliferative activity, whereas those in the indeterminate nodules lose it (and, hence, the proliferative potential) at early steps of development [145,146].

Differentiation of rhizobia into bacteroids can be either reversible or irreversible, depending on the type of nodules [142]. The type of the implemented developmental program depends on the ability of the root nodules to promote the terminal differentiation—irreversible transformation of the bacterial cells into bacteroids [147]. Currently, nodule-specific cysteine-rich (NCR) peptides are recognized as the key players in the regulation of the terminal differentiation. These molecules are synthesized by the the legume species representing the inverted-repeat-lacking clade—a monophyletic group of the flowering plant subfamily *Faboideae* (or *Papilionaceae*), including most of the legume crops [147,148]. Thereby, each species has a broad selection of the NCR peptides of essentially different diversity [149] (from seven in *Glycyrrhiza uralensis* Fisch. ex DC., to over 600 in *M. truncatula* [147]), which are involved in the terminal differentiation at multiple steps [147]. The sequences of NCR peptides typically contain 60–90 amino acid residues, including conserved N-terminal signal domains, and the mature peptides form disulfide bonds due to the presence of four or six cysteine residues. Despite some similarities in structure (e.g., occurrences of characteristic sequence moieties and repeats), the sequences of NCR peptides appear to be highly variable and heterogeneous [150]. Usually, cationic, anionic, and neutral NCR peptides are distinguished [147].

Depending on their sequences and pKa values, the NCR peptides can possess relatively high antimicrobial activity, which makes them attractive as potential antimicrobial agents [149]. Initially, Mergaert et al. identified over 300 different sequences of genes encoding NCR peptides in *Medicago truncatula*, *Pisum sativum*, *Vicia faba* L., and *Trifolium repens* L. [150]. Recently, the authors extended the list of characterized NCRs to 600 with new members of this peptide family found in *Glycyrrhiza uralensis*, *Oxytropis lambertii* Pursh, *Astragalus canadensis* L., *Onobrychis viciifolia* Scop., *Galega orientalis* Lam., *Ononis spinosa* L., *Cicer arietinum* L., and *Medicago sativa* [147]. Additionally, Zorin and colleagues found 360 genes encoding NCR peptides that are expressed in nodules of *Pisum sativum* [151]. 

The biological role of the theoretically predicted peptides was confirmed experimentally. Van de Velde et al. showed that NCRs penetrate the bacterial plasma membrane and induce differentiation of rhizobia into bacteroids in the cytoplasm of root cells [152]. Presumably, NCR peptides regulate the levels of the proteins involved in the regulation of the cell cycle: CtrA, DnaA, GcrA, and FtsZ [153,154,155]. DnaA initiates replication of the prokaryotic DNA and activates GcrA, which, in turn, represses DnaA and upregulates CtrA [156,157]. The CtrA protein acts at the late stage of the cell cycle to repress initiation of replication and promote cell division [155,156]. Not less importantly, NCRs affect the activity of FtsZ, involved in the formation of the Z-ring [154,157]. Therefore, activation of DNA replication and inhibition of cell division promotes the differentiation of rhizobia into bacteroids.

On the other hand, individual NCR peptides can act as positive regulators for one legume species and negative ones for others [158]. This seems to be an important factor underlying the specificity of the symbiosis. The experimental disruption of the NCR transport to bacterial cells inhibited terminal differentiation of rhizobia in bacteroids [152]. However, the ectopic expression of *NCR* genes in the legume species not belonging to the inverted-repeat-lacking clade (and, therefore, not synthesizing NCRs constitutively) induced the symptoms of terminal differentiation [152]. Analysis of the tissue distribution of NCRs in the indeterminate nodules revealed their predominant localization in the nitrogen-fixing zone.

## 7. Autoregulation of Nodulation

High relative contents of nitrogen-rich proteins in the storage tissues are one of the most important biochemical features of the legume seeds [159]. This supply ensures successful germination and supports seedlings during the early stages of their development [160]. Due to this supply, the seedlings can grow and preserve their photosynthetic activity before the switch to external sources of nitrogen [161]. As establishment and maintenance of symbiosis are energy consuming, this metabolic feature of legume seeds and seedlings is a critical prerequisite for successful rhizobial inoculation [23]. Depletion of the seed storage results in development of nitrogen starvation, which acts as a trigger for the signaling events leading to rhizobial colonization. Indeed, reduced nitrogen availability can be percepted by plants—it was shown to initiate nodulation, while the normal nitrogen supply inhibits it [162].

A new class of plant hormone-like C-terminally encoded peptides (CEP) involved in growth and development of the legume root system was discovered [163]. It can be assumed that these molecules play a key role in the initiation of the nodule formation. To date, more than 15 individual CEPs are characterized. All representatives of this family share a common sequence moiety of 15 amino acid residues with one or several characteristic proline residues, which typically demonstrate complex and dynamic patterns of hydroxylation [164,165]. Thereby, most of the CEP peptides can be attributed to one of the two groups with characteristic sequences D/A/E-F-A/R-P-T-N/T/S/E-P/Q-G/E-H/N/D/P-S/N-P/Q/S-G-I/VV/M-G/R/H and I/V-Y/D-R-R/Y-L/Q-E/G/R-S/D-V-P-S-P-G-V/I-G-H [164]. Interestingly, replacing the glycine residue at the eighth position with alanine cancels CEP-induced inhibition of the lateral root formation [166]. Recently, Roberts et al. showed that in *Arabidopsis thaliana*, the genes encoding CEP peptides (CEP1, CEP3, CEP4, CEP5, CEP9, CEP13, CEP15) are predominantly expressed in roots, although some lower expression levels of all these peptides can be detected in all parts of the shoots, and that this type of peptides involved in signaling is found only in angiosperms and is not present in evolutionarily older taxons [164]. This might indicate a more universal physiological role of these peptides than is currently assumed.

**Figure 5:**
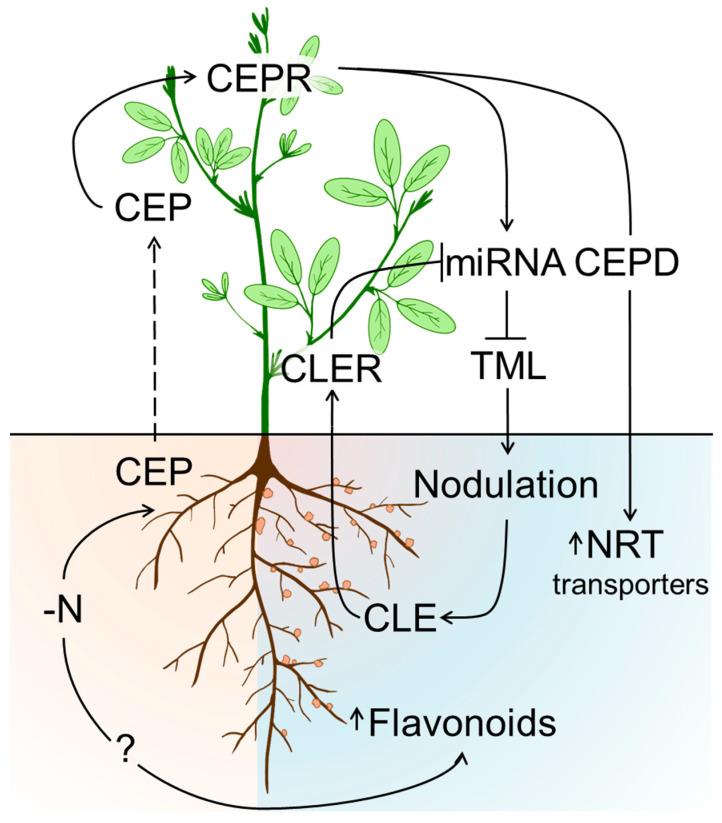
The role of the C-terminally encoded peptides (CEP) and CLAVATA3/Embryo-surrounding region-related peptides (CLE) in induction of nodulation. CEPR—C-terminally encoded peptide receptor; CLER—nodule autoregulation receptor kinase (GmNARK/LjHAR1/MtSUNN/PsSYM29); CEPD—C-terminally encoded peptide downstream; NTR—nitrogen root transporters; miRNA—microRNAs or non-coding RNA molecules; TML—regulator of transcription.

The CEP peptides attracted a special interest when, in the middle of the last decade, Tabata et al. showed that the CEP-associated signaling correlates well to the contents of metabolically available nitrogen in soils [165]. Later on, Ohkubo et al. showed that interaction of CEPs with their receptor (CEPR) leads to the synthesis of C-terminally encoded peptide downstream (CEPD) types one and two [167]. These signaling peptides were synthesized in leaves and readily migrate to the root and increase the expression of nitrogen transporters in the root cells [167]. Based on the correlation between nitrogen levels in soils and synthesis of the CEP peptides *in planta* [13], it was logical to assume the involvement of CEPs in the formation of the legume–rhizobia symbiosis [167]. Thus, Imin et al. showed for *Medicago truncatula*, *M. sativa*, *Trifolium subterraneum*, and *T. repens* that the over-expression of *MtCEP1,* or the addition of its synthetic analog to the growth medium, increased the numbers of the nodules formed, and enhanced nitrogen fixation even in presence of high nitrate concentrations in the medium (i.e., the conditions, which typically suppress nodulation) [166]. It is important to note that root hair swelling and enhanced nodulation were equally pronounced in presence or in absence of rhizobia (in the absence of bacteria, spontaneous empty nodules formed). This might indicate that reduction in nitrogen uptake triggers the synthesis of CEP peptides in roots. The synthesized peptides migrate to the leaves and bind to the CEPR receptor. After this, CEPR activates microRNA miR2111, which migrates to the root and negatively regulates its targets—TML1/2 (*Too Much Love 1/2*, the inhibitors of nodulation), that results in induction of nodulation [168] (Figure 5).

Due to the large energy demand of the nodule metabolism, the plant can afford only limited numbers of completely developed nodules. This aspect is regulated by CLAVATA3/Embryo-surrounding region-related peptides (CLE, Figure 5) [169]. Therefore, once nodulation is induced, the probability of re-infection of the same root is rather low. The enhanced expression of CLE leads to suppression of the nodulation signals [136,137]. Thus, these peptides are involved in regulation of nodule formation and essentially impact on the nodulation efficiency. The sequences of the CLE peptides are rather conservative, although they are widely spread in the plant kingdom and are featured with high functional diversity [170]. To date, 33 CLE peptides are characterized for *Arabidopsis thaliana*, five for *Oryza sativa* L., two for *Zea mays* L., and one for *Solanum lycopersicum* L. [170]. Based on their sequence, these peptides can be split into two groups, usually referred to as CLE-A and CLE-B [170]. The N-terminal amino acid residue of the A-type peptides is always arginine. The generalized sequence of this major class of CLE peptides can be summarized as R-L/V-V/I-P/H-X-G-P-N/D-P-L/I-H-N/H [171]. The group of the B-type peptides is much less diverse and can be represented with the generalized sequence H/R-X-X-X-S-G/P-N/D-R/P-L/I-S/H-N [171]. The over-expression of the *CLE* genes was shown to inhibit nodulation [136]. The nodulation competence could be restored by a knock-down mutation of the CLE peptide receptor, as was shown for *L. japonicus* [172]. The CLE peptides are transported to the shoot through the xylem and interact with the nodule autoregulation receptor kinase (GmNARK/LjHAR1/MtSUNN/PsSYM29) [173]. Interaction of CLEs with their receptor causes inactivation of miRNA miR-2111 and, therefore, inhibition of nodulation by TML1/2 [174,175]. Thus, CEP and CLE peptides control the autoregulation of nodulation through control of the miRNA/TML levels (Figure 5).

Similar mechanisms are involved in the control of arbuscular mycorrhiza development. For example, a mutation in the gene encoding CLER promotes both legume-microbial interactions—on one hand, it leads to the formation of excessive numbers of root nodules, and on the other, it induces hypermycorrhization [176,177,178]. CLE peptides are also involved in the suppression of mycorrhizal colonization. Moreover, the control of the arbuscular mycorrhizal colonization by CLE peptide signaling appeared to be very conservative across a broad range of legume and non-legume species [178,179,180]. Thus, along with the early signaling interactions that lead to the formation of nodules and arbuscular mycorrhiza, autoregulation might also act as a general symbiotic toolkit that allows plants to control the development of the symbiont.

## 8. Nodule Senescence

Senescence is the final stage in the nodule life and in development of the legume–rhizobia symbiosis [181]. At the biochemical level, it is accompanied with dramatic changes in the nodule metabolism. Firstly, senescence is accompanied with the changes in the nodule color, which turns from pink to green due to degradation of leghemoglobin [182]. Further, symbiosomal membranes disintegrate, and the enclosed molecules are released simultaneously with the activation of proteolytic enzymes [183,184]. In determinate nodules, visual signs of senescence develop radially starting from the central part of the nodule and then slowly spreading outwards [185]. In contrast, a morphologically distinguished senescence zone is formed in the indeterminate nodules [185]. During the nodule development, this zone gradually migrates in the distal direction until it reaches the apical part of the nodule that indicates the transition to the stage of the nodule degeneration [185].

Despite dramatic phenotypic changes accompanying nodule senescence, this process is the least studied with respect to the underlying signaling mechanisms and pathways. It is clear, however, that, similarly to other stages of symbiosis, nodule senescence requires coordinated interaction of the bacterial partner and the host plant. Based on the transcriptomics data acquired for the bacteroids formed by *Bradyrhizobium diazoefficens* in soybean nodules, Franck et al. showed that, depending on the developmental stage and environmental factors, the bacteroids demonstrated essential variations in gene expression profiles (which were manifested with increased, decreased, and/or patterned transcription of individual genes) during nodulation, after nodule formation, and in senescence [186]. It means that all stages of a nodule’s life cycle, in particular senescence, were controlled by the plant.

Usually, the onset of senescence occurs at late stages of the legume development when flowering is completed typically. According to this generalized scenario, the accompanying patterns of anatomy changes and metabolic shifts develop in parallel to seed formation and maturation [187]. However, it needs to be taken into account that application of environmental stress dramatically accelerates the onset of senescence and might enhance manifestation of all accompanying changes in nodule morphology, anatomy, physiology, and metabolism [188,189].

In general, plant senescence and response to environmental stress are accompanied by generation of reactive oxygen species (ROS) in plant cells and development of oxidative stress [190,191]. On the other hand, the important factor of the senescence onset is the age-related shift in the nodule redox status. Thus, Ivanova and co-workers showed that a functional metabolically active nodule requires high amounts of reduced glutathione (GSH) in tissues, whereas accumulation of its oxidized form (GSSG) correlated with nodule senescence [192]. Fukudome et al. also found that the nodule content of nitric oxide (NO) is one of the key regulators of nodule senescence. The suppression of NO accumulation by over-expression of LjGlb1 (class 1 phytoglobin) in *Lotus japonicus* resulted in prolonged nodule function, while treatment with 1-aminocyclopropane-1-carboxylic acid (ACC, ethylene precursor) resulted in inhibition of nitrogenase and induced expression of senescence genes [193]. Based on the observation that legume root nodules undergo accelerated aging under stress conditions, it seems likely that this process is triggered and at least partly coordinated via ROS-signaling [194]. However, the signal cascades and regulatory mechanisms involved in the control of nodule senescence are still unknown.

It is important to note that aging of root nodules is also accompanied by enhanced lipo- and glycoxidation [195]. Indeed, the age-related enhancement of oxidative stress occurs at the background of high sugar concentrations present in nodules. This aspect was confirmed by analyzing the proteome and metabolome of nodules [196,197]. Thus, the age-related increase in protein glycation and lipoxidation can be expected in all plant organs including root nodules [196,197,198]. It needs to be taken into account that due to high glycation potential of regulatory sugars and sugar-related molecules (fructose, sucrose, poly(ADP-ribose), sugar phosphates) [199], it can be assumed that glycation might be involved in regulation of plant stress response [198]. Most of the genes involved in the nodule senescence encode cysteine proteases, transcription factors, regulatory peptides, and membrane proteins [200]. Of course, glycation can affect the structures of these proteins and thereby disrupt their functions. Moreover, in a similar way, it can affect the proteins involved in hormone signaling. 

So far, the phenomenon of plant senescence is most intensively studied for leaves. The leaf senescence is tightly regulated with phytohormones—ethylene, salicylic acid, abscisic acid, and jasmonates impact the senescence onset and accompanying metabolic rearrangements associated with this process [201,202,203,204]. Most likely, to some extent, similar hormone-mediated mechanisms are involved in the senescence of legume root nodules. Indeed, in their work on the transcriptome of *Medicago truncatula* nodules, Van de Velde and co-workers reported age-related alterations in expression of corresponding genes. Thus, the expression of the genes of ethylene biosynthesis and ethylene-dependent transcription factors was up-regulated. The same was the case for the genes of lipoxygenases involved in the initial stages of jasmonate biosynthesis [185]. Interestingly, the authors did not find any increase in the expression of the genes involved in the biosynthesis of abscisic acid [185]. On the other hand, Serova and co-workers showed that gibberellins prevented nodule senescence [205]. Altogether, these facts confirm the key role of phytohormones in the regulation of legume nodule senescence, but the underlying molecular mechanisms still remain unknown.

## 9. Conclusions

Legume–rhizobia symbiosis is highly (if not critically) important for survival and productivity of legumes. Therefore, legume–rhizobia symbiosis has been comprehensively studied for decades. However, despite impressive progress, some regulatory aspects of nodule development and the signaling pathways behind them are still insufficiently addressed. In particular, a better understanding of the molecular mechanisms underlying stimulation of flavonoid biosynthesis in response to nitrogen starvation, as well as the transport of flavonoids in the rhizobial cell, could yield new strategies to increase the efficiency of legume–rhizobia symbiosis. In this context, a study of exact signaling mechanisms and all molecular players involved in the first interaction of rhizobia with the plant cell is very important. Not less importantly, for some rhizobial species, the structures of Nod factors remain unknown. On the other hand, the receptors for Nod factors were reported in several legume species, but other homologous receptors, especially receptors for bacterial exopolysaccharides, are still not sufficiently addressed. The new knowledge about the signaling pathways and the role of phytohormones in nodule formation can help with development of new strategies for development of legume crops productivity. Another promising direction of further research would be clarification of the exact mechanisms behind the release of rhizobia from the infection thread into the cytoplasm of the legume cell. Not less importantly, although the changes in the metabolism and the biochemistry of nitrogen fixation in bacteria during their transformation into bacteroids have been studied, the signaling events triggering these changes are still not identified. In addition, while the NCR peptides promoting differentiation of rhizobia into bacteroids in indeterminate nodules have been intensively studied, signaling molecules for the determinate ones have not yet been found. Furthermore, the research of signaling mechanisms behind the triggering of nodule senescence and development of the related stress response will help identify new ways for development of plant protection approaches.

## Abbreviations

ACC	1-aminocyclopropane-1-carboxylic acid
ADP	Adenosine diphosphate
ATP	Adenosine triphosphate
CBS1	Cystathionine-β-synthase-like 1
CCaMK	Calcium/calmodulin-dependent serine/threonine-protein kinase
CEP	C-terminally encoded peptide
CEPD	C-terminally encoded peptide downstream
CEPR	C-terminally encoded peptide receptor
CEPs	C-terminally encoded peptides
CLEs	CLAVATA3/Embryo-surrounding region-related peptides
CRE1	Cytokinin receptor 1
FA	Fatty acid
GSH	Reduced glutathione
GSSG	Oxidized form of glutathione
IPD3	Interacting Protein of DMI3 of *Medicago truncatula* (homolog of the protein CYCLOPS of *Lotus japonicus*)
IT	Infection thread
MLD	Malectin-like domain
NCR	Nodule-specific cysteine-rich peptide
NFR1	Nod Factor Receptor 1 kinase
NFR5	Nod Factor Receptor 5 kinase
NFs	Nod factors
NF-Y	Nuclear factor Y
NiCK 4	NFR5-interacting cytoplasmic kinase 4
NIN	Nodulation inception transcription factor
NO	Nitric oxide
NodA	Acyl transferase
NodB	Deacetylase
NodC	N-acetyl glucosamine transferase
NodD	Nodulation protein D
NodM	Glucosamine synthase
Nop	Nodulation outer proteins
NPL	Nodulation pectate lyase
NTR	Nitrogen root transporters
ROS	Reactive oxygen species
RPG	Rhizobium-directed polar growth protein
SAR	Structure activity relationships
SYMRK	LRR-containing receptor kinase
T3SS	Type III secretory system
T4SS	Type IV secretory system
VPY	Vapyrin
